# Imaging built-in electric fields and light matter by Fourier-precession TEM

**DOI:** 10.1038/s41598-024-51423-x

**Published:** 2024-01-15

**Authors:** Tizian Lorenzen, Benjamin März, Tianhao Xue, Andreas Beyer, Kerstin Volz, Thomas Bein, Knut Müller-Caspary

**Affiliations:** 1 Department of Chemistry and Center for NanoScience, Ludwig-Maximilians-Universität München, Butenandtstr. 11, 81377 München, Germany; 2 Louisiana State University Shared Instrumentation Facility (LSUSIF), 121 Chemistry and Materials Building, 4048 Highland Rd., Baton Rouge, LA 70803 USA; 3 Department of Physics, Philipps University Marburg, Hans-Meerwein-Straße 6, 35032 Marburg, Germany

**Keywords:** Transmission electron microscopy, Semiconductors

## Abstract

We report the precise measurement of electric fields in nanostructures, and high-contrast imaging of soft matter at ultralow electron doses by transmission electron microscopy (TEM). In particular, a versatile method based on the theorem of reciprocity is introduced to enable differential phase contrast imaging and ptychography in conventional, plane-wave illumination TEM. This is realised by a series of TEM images acquired under different tilts, thereby introducing the sampling rate in reciprocal space as a tuneable parameter, in contrast to momentum-resolved scanning techniques. First, the electric field of a *p–n* junction in GaAs is imaged. Second, low-dose, in-focus ptychographic and DPC characterisation of Kagome pores in weakly scattering covalent organic frameworks is demonstrated by using a precessing electron beam in combination with a direct electron detector. The approach offers utmost flexibility to record relevant spatial frequencies selectively, while acquisition times and dose requirements are significantly reduced compared to the 4D-STEM counterpart.

## Introduction

In recent years, nanostructured materials have come increasingly into the focus of research in the fields of information and energy technology. Porous organic materials with highly ordered structure and tunable functionalities, such as metal and covalent organic frameworks (MOFs/COFs) are investigated for their optoelectronic properties or applications in energy storage, catalysis and gas storage^1,2^. Halide based perovskites are explored for their applications in solar cells and lasers^3^. The functional properties of such devices are fundamentally determined by the structure, i.e., the nanoscale particle shapes, pores and atomic configuration. Understanding the structure–property relationships is central when designing applications and searching for suitable candidate materials. In addition to deciphering the structure via transmission electron microscopy (TEM) with a spatial resolution down to a few tens of picometres, mapping the small built-in electric fields in semiconductor nanostructures such as *p–n* junctions^4^ remains a severe challenge for TEM.

Conventional TEM imaging of light atoms in organic chemistry or structural biology always involves some form of compromise. Unfortunately, weakly scattering specimens show no image contrast in the absence of aberrations at zero defocus as they only shift the phase of the illuminating electron wave slightly. In these cases, deliberately introducing partly large aberrations through defocusing is effective in converting phase shifts into amplitude contrast. While this is widely accepted for improving contrast, it comes at the expense of image resolution and complicates direct interpretability. On top of that, organic and biological specimens are highly dose-sensitive, making trustworthy structural imaging with a dose budget in the range of ten electrons per Å$^2$ very complicated. This is approximately three orders of magnitude less than in typical materials science applications.

In the last decade efficient phase contrast generation has been developed in scanning TEM (STEM) by increasing the dimensionality of acquired data using segmented or pixelated detectors leading to 4D-STEM. In essence, 4D-STEM aims at collecting complete diffraction patterns at preferably high spatial frequency sampling, for each raster position $(r_x, r_y)$ of the electron probe in real space thereby leading to 4D-data sets. Differential phase contrast^5–7^ (DPC) or centre-of-mass (COM) imaging^8^, as well as a variety of ptychographic methods^9–11^ have proven to be effective phase contrast methods. Successful applications include the mapping of atomic and mesoscale electric^12^ and magnetic fields^13^, and high-contrast imaging of light matter at low electron dose^14^. Substantial efforts are currently put into the technological and conceptual development of multisegment DPC^15^ or pixelated detectors^16–18^, and into coping with the resulting tremendous data rates. Yet, today, fast STEM detectors still only have a limited number of segments while large pixelated detectors with $10^4$–$10^5$ pixels are comparatively slow. This means that currently suitably large fields of view at sufficient real space samplings can only be achieved with DPC detectors with a few segments at most.

**Figure 1:**
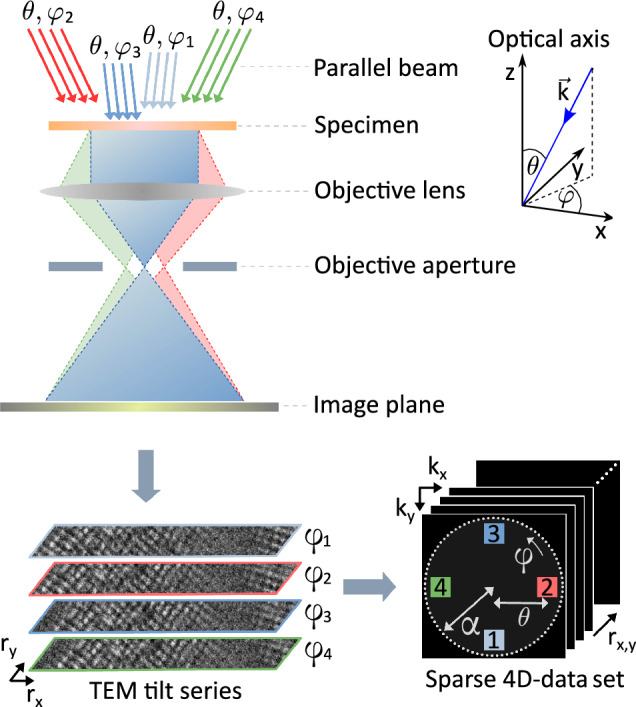
Experimental setup for Fourier TEM acquisitions of STEM DPC data. A multitude of TEM images with differently tilted parallel illumination is recorded to generate a set of sparse diffraction patterns (DPs). The bright field disk is limited by the radius of the objective aperture $\alpha$, each beam tilt is characterised by its polar angle $\theta$ and azimuth $\varphi$ relative to the optical axis. The used coordinate system is given in the top right.

In this work, motivated by the requirement of large-scale electric field mapping and high-contrast imaging of light matter, a technique to overcome these limitations is developed conceptually and in applications. Based on the theorem of reciprocity in optics, we demonstrate the imaging of built-in electric fields in a *p–n* junction, and the enhancement of low-dose image contrast in organic nanostructures, such as a COF. The kernel of the method involves the acquisition of sparse 4D data using conventional plane-wave illumination TEM to record real space images for different tilts of the incident electron beam. This is schematically shown in Fig. 1 and importantly maintains the large field of view of TEM. In the general field of microscopy including light-optical ptychography, this acquisition scheme is occasionally referred to as Fourier ptychography^19^. The obtained data is then subjected to advanced 4D-STEM evaluations such as DPC, COM and a variety of ptychographic algorithms. By combining the precession capability of a conventional TEM with an ultra-fast camera, acquisition of DPC data with 100 segments is demonstrated, for which otherwise 4–16 segments are currently common. In addition, the method overcomes the hardware-dictated sampling of diffraction patterns, restricts the electron dose to recording only those spatial frequencies that are expected to carry the most relevant information about the specimen, and does not suffer from hydrocarbon contamination arising from focused probes.

**Figure 2:**
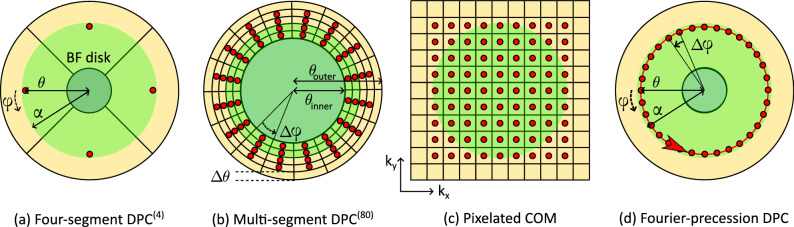
Enabling arbitrary STEM detector geometries by Fourier TEM. Schematic geometries of detectors (yellow), corresponding patterns of beam-tilt series (red) and the bright-field region limited by the aperture radius $\alpha$ (green) in the backfocal plane of a STEM. (**a**) Standard four-segment differential phase contrast ($\textrm{DPC}^{(4)}$) detector, (**b**) theoretical multi-segment $\textrm{DPC}^{(80)}$ detector with inner and outer collection angle $\theta _{\textrm{inner}}$ and $\theta _{\textrm{outer}}$ consisting of eighty segments, both approximated by one beam tilt for each segment (characterised by polar angle $\theta$ and azimuth $\varphi$ with the corresponding spacing of $\Delta \theta$ and $\Delta \varphi$), (**c**) pixel array detector where each detector pixel equates to one tilt (rectangular pattern with reciprocal space vectors ${k_x}, {k_y}$) for COM and 4D-STEM. (**d**) Fourier-precession DPC recorded with a beam continuously precessing around the optical axis. The central areas in (**a**, **b**, **d**) represent holes in hardware detectors.

Following the optical theorem of reciprocity, mapping the intensity of a diffraction coordinate $\vec {k}_\perp$ in the STEM Ronchigram against the scan position is identical to recording a TEM image under plane wave illumination, tilted such that the lateral component of the wave vector equals $\vec {k}_\perp$^20–22^. Considering an arbitrary geometry of STEM detectors in the bright field (BF), as exemplified in Fig. 2, the STEM signal of each detector segment can as well be obtained by a single or a series of plane wave illumination TEM images with appropriate beam tilts. By solely sampling spatial frequencies according to the red dots in Fig. 2a–c, four-segment $\textrm{DPC}^{(4)}$, 80-segment $\textrm{DPC}^{(80)}$ with five rings, and COM imaging can be realised without investing dose in the irrelevant dark green regions. The superscript notation is used to denote the number of segments of the fictitious DPC detector. Accordingly, this also allows the realisation of detectors that are currently unavailable or technically unfeasible, such as a 100-segment DPC detector which we emulated by using a precessing beam together with an ultrafast camera (Fig. 2d). In both STEM and TEM the bright field disk radius is defined by an aperture with radius $\alpha$, which equals the semi-convergence angle in STEM and the radius of the objective aperture in TEM.

Whereas the choice whether to use 4D-STEM or a TEM tilt series to acquire the data appears neutral from the abstract physical point of view, the latter offers drastic enhancement of the sensitivity to beam deflections due to small electric fields, and the dose sensitivity in weakly scattering objects. This is because the beam tilt can be controlled continuously in a TEM setup in contrast to a detector with fixed pixel size in STEM. The fixed detector size leads to the requirement of huge camera lengths of up to several hundred meters with correspondingly small convergence semi-angles when imaging extremely small fields^23^. Most importantly, DPC measurements of organic and biological specimens^14^ employ segmented ring detectors with a central hole to detect shifts of the undiffracted beam. Although the central hole in the detector is justified because outer Ronchigram parts dominate the DPC contrast, electrons passing through the hole cause specimen damage without contributing to the signal.

For the direct analysis of the obtained data sets the differential phase contrast (DPC) method was used as it offers a physical interpretation of the obtained signal^8^. The DPC vector field can be interpreted as the (probe-convolved) electric field^24^ if dynamical scattering and propagation in the specimen can be neglected, and the field does not vary at the scale of the probe. It can be integrated to obtain the approximate projected potential of the specimen, leading to integrated differential phase contrast^25^ (iDPC). Following reciprocity, more involved ptychographic algorithms such as single-sideband^10^ (SSB) reconstructions are applied to data sets from TEM tilt series as well. In particular, they allow for an *in silico* correction of imperfections of the electron-optical system, such as defocus and the spherical aberration.

## Results

### Detection of built-in electric fields

**Figure 3:**
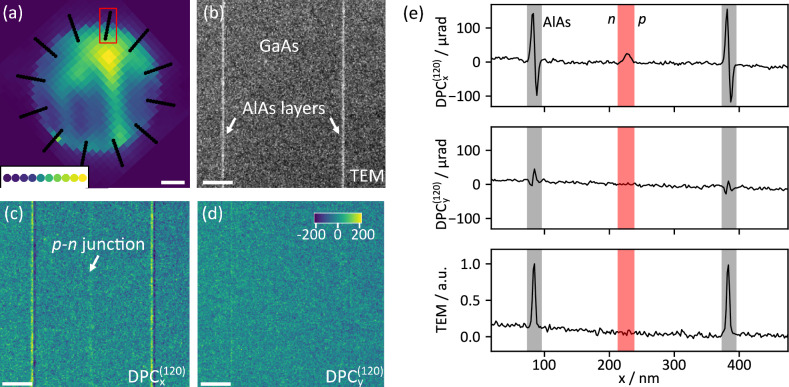
Fourier DPC imaging of electric fields in a *p–n* junction. (**a**) Tilt pattern of 120 tilts used in the imaging of the *p–n* junction schematically superimposed to a PACBED obtained using a square tilt pattern of 31 by 31 tilts. The inset shows a line of tilts (red box) from the recorded data set coloured according to the averaged intensity. The length of the scale bar is 1 mrad. (**b**) Conventional TEM image of a GaAs specimen with an invisible *p–n* junction, centred between two AlAs marker layers. (**c**) The *p–n* junction is clearly visible in the *x*-component of the obtained DPC signal. (**d**) In the *y*-component the *p–n* junction is invisible. The colourbar in (**d**) applies to plots (**c**) and (**d**) and denotes the local shift in µrad, the length of the scalebar equals 75 nm. (**e**) Averaged DPC shifts of the *x*- and *y*-components (top, middle) and the TEM intensity (bottom, in arbitrary units) are shown. The area of the *p–n* junction is highlighted in red, the AlAs layers are marked in grey.

A *p–n* junction in a GaAs compound semiconductor was analysed by Fourier DPC, as documented in Fig. 3. The small electric fields only cause tiny intensity redistributions within and close to the rim of the bright field disk. The imaging of *p–n* junctions is therefore hardly achievable with standard electron microscopy techniques such as bright field TEM and dark field STEM. Dedicated phase contrast techniques have been developed for this purpose, usually employing DPC^26^ or pixelated^4,27^ detectors for COM imaging and disk detection^28^, by using electron holography^27,29^ or using iterative phase retrieval techniques such as diffractive coherent imaging^30^. Assuming a constant electric field across the interaction volume, a linear relation between average momentum transfer and electric field applies^8,24^.

Two different tilt patterns have been applied as depicted in Fig. 3a. First, a cartesian pattern of $31\times 31$ beam tilts across an objective aperture with a radius of 2.8 mrad has been used for reference and demonstrative purposes of reciprocity. The position-averaged convergent-beam electron diffraction pattern (PACBED), consisting of mapping the average TEM intensity at each beam tilt against the tilt coordinate, is shown colour-coded in Fig. 3a. The intensity variations inside the PACBED arise from slight orientation- and thickness gradients within the imaged region shown in Fig. 3b. Whereas the cartesian tilt pattern allows a true COM calculation from the BF region which indeed shows the *p–n* junction midway between the AlAs markers with excellent contrast (Suppl. Fig. 1), the recording of $31^2=961$ TEM images through software scripting takes a significant amount of time at the order of 15 min. This is unfavourable in practice as such long measurements are strongly affected by specimen drift.

Thus another sampling strategy was realised so as to approximate the centre-of-mass measurement efficiently. The COM concept relies on weighting diffraction intensities proportionally to the scattering angle. Therefore, low scattering angles within the bright field disk make a minor contribution to the COM signal. The same is true for scattering angles just beyond the BF disk, because the intensity itself drops drastically at the transition to the dark field. At the same time, spatial frequencies at the rim of the BF disk are subject to extremely faint intensity redistributions in the presence of small electric fields, suggesting the use of a high diffraction space sampling within just this scattering angle range. Therefore, a tilt pattern representing a multi-segment detector was used as shown by the black dots in Fig. 3a, utilising a high sampling in those regions most sensitive to beam deflection, while reducing the total number of acquisitions by nearly an order of magnitude. An acquisition scheme of 120 tilts arranged in 10 circles with 12 tilts each was applied, leading to a Fourier DPC signal termed $\textrm{DPC}^{(120)}$. Note that this corresponds to a DPC detector with 120 segments. The inner circle started at a radius corresponding to 0.8 times the BF disk, that is, objective aperture radius $\alpha$. Consecutive circles reach slightly into the dark field with an outer radius of $\theta _{\textrm{outer}} = 1.2\cdot \alpha$. Using this sampling strategy the influence of drift was eliminated due to the accelerated acquisition. As expected the *p–n* junction remains invisible in the TEM bright-field shown in Fig. 3b but is clearly detected in the DPC$^{(120)}_x$ component in Fig. 3c. Due to the horizontal orientation of the field vectors, it is invisible in the *y*-component plotted in Fig. 3d.

Because of the 1D geometry of the data in Fig. 3, the signals have been averaged vertically along the *p–n* junction. The resulting 1D-profiles for the *x*- and *y*-components of the DPC$^{(120)}$ signal, as well as the TEM intensity are shown in Fig. 3e. The *x*-component visualises jumps of the mean inner potential (MIP) at the AlAs marker interfaces causing the largest deflection of the probe around 100 $\upmu$rad, being strongly affected by dynamical scattering^31,32^. Importantly, the angular deflection caused by the built-in electric field is clearly visible above the noise level (by a factor of 10) mid-way between the AlAs layers and amounts to approximately 25 $\upmu$rad. This feature is solely present in the DPC$^{(120)}_x$ plot, but not in the DPC$^{(120)}_y$ and TEM profiles below. The small slope in both the TEM signal and the DPC$^{(120)}_y$ component can be assigned to small thickness and crystallographic tilt gradients.

The total depletion region of the *p–n* junction approximates 26 nm with a full width at half maximum (FWHM) of 17 nm, which is in good agreement with values reported previously^4^. The angular deflection of 25 $\upmu$rad translates into a projected electric field magnitude of 8.6 V, having the unit field times thickness. However, both preparation-induced electrical passivation of surface layers leading to an imprecisely known electrically active thickness of the specimen and dynamical scattering hinder the quantification of the electric field strength in terms of its projection average.

### Fast imaging of covalent organic frameworks

To enable in-focus, high-resolution phase contrast imaging of beam sensitive specimens, such as covalent organic frameworks, the acquisition speed was further improved drastically. Two major modifications were made to the approach presented above. First, the discrete tilting using the software interface was circumvented by conducting the TEM acquisition at hollow-cone illumination^33^. In this illumination mode, the incident beam is precessing at constant inclination $\theta$ while $\varphi$ is changing continuously. Second, we employed an $256\times 256$ ultra-fast Medipix3 direct electron detector, which is capable of kHz rate acquisitions^16,34^. In order to synchronise precession and TEM acquisition, an electronic trigger circuit was developed which assures that the azimuthal sequence of TEM images remains constant among different recordings, and that image acquisition and precession remain in phase when many precession cycles are recorded. According to Fig. 2d, dose is only invested in the explicit scattering angles on the red circle close to the rim of the bright field disk. With continuous read-out of a camera at kHz rate without dead times between subsequent images, the azimuthal sampling easily corresponds to a DPC$^{(100)}$ or DPC$^{(1000)}$ detector in STEM.

Here a dibenzo[g,p]chrysene (DBC)-based covalent organic framework (COF) of highly crystalline nature was studied, which is known to be both a beam-sensitive as well as a weakly scattering material. Similar to other 2D COFs^35^, the adjacent two-dimensional layers are stacked in the z-direction due to $\pi$–$\pi$ interactions between aromatic systems. The structure consists of vertical channels forming a Kagome lattice, an important structural feature for potential applications in the field of energy materials or in optoelectronics^36,37^. In this experiment, the semi-angle of the precession cone opening was chosen to be $\theta =0.98\cdot \alpha$ with an objective aperture radius of $\alpha =6\,$mrad. Within a single precession period of 1 s a total of 100 acquisitions were performed, each with an exposure time of 10 ms. This resulted in nominal precession steps of $\Delta \varphi = {3.6}^{\circ }$, corresponding to a DPC$^{(100)}$ detector. A dose of 33 electrons per Å$^2$ was measured on the detector, multislice simulations suggest that the dose at the specimen should be at most twice as high given the thickness-dependent cutoff of scattered electrons by the objective aperture (see Suppl. Sect. 1 and Suppl. Fig. 2).

**Figure 4:**
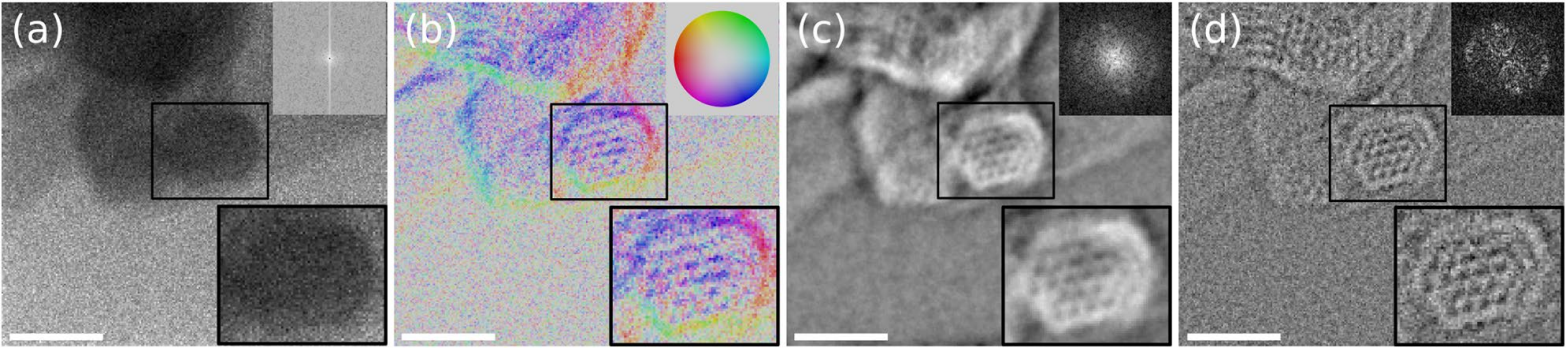
Fourier-precession TEM imaging of a covalent organic framework by DPC and ptychography. (**a**) ABF, (**b**) DPC vector field, (**c**) iDPC signal and (**d**) ptychographic reconstruction of a covalent organic framework obtained using precession DPC. The top right insets in (**a**, **c**, **d**) show the power spectra, and in (**b**) the colour wheel (in arbitrary units). The bottom right insets show the zoomed-in central Kagome lattice. The scalebar represents 50 nm.

From this data, annular bright field (ABF), vectorial DPC and scalar integrated DPC (iDPC) images can be created straight forwardly, whereas the drastically enhanced segmentation also paves a practical way for ptychography^38^ based on a DPC setup. The different signals for the COF are shown in Fig. 4. The ABF image in Fig. 4a was obtained by a summation of the image series of one precession cycle, without further processing. Obviously, only the coarse shape of the particles, but no pores or COF channels are resolved. This was confirmed by the corresponding power spectrum, not showing any sign of periodicities. The obtained DPC$^{(100)}$ vector field is shown in Fig. 4b, with vector magnitude and direction coded by hue and colour according to the colour wheel inset, respectively. At the edges of the COFs, the DPC$^{(100)}$ vectors indicate a deflection of the electrons towards the particles. This is expected, since the electron waves are refracted in the direction of increasing mean inner potential. Additional lens aberrations such as defocus and spherical aberration can enhance this and result in fringes at the COF edges as shown in Suppl. Fig. 3. Note that the COF channels are clearly visible in Fig. 4b, albeit slightly noisy.

By integrating to the iDPC$^{(100)}$ signal, noise is inherently reduced^6,25^ as seen in Fig. 4c. It reflects the phase that would be imprinted on an incident plane wave in TEM imaging, and which would usually be lost in the recording process in the absence of aberrations and defocus. While the iDPC signal and the specimen potential are only proportional to each other in ultimately thin specimens (within the limits imposed by the transfer function), image simulations show that the method is rather robust with respect to specimen thickness and defocus as demonstrated in Suppl. Figs. 4 and 5. Apart from the improved contrast, the iDPC$^{(100)}$ signal in Fig. 4c clearly resolves the Kagome structure of the framework, consisting of corner-connected hexagonal channels with spacings of approximately 6 nm.

In addition, the same data set was used to perform a single-sideband ptychography reconstruction^10^ as shown in Fig. 4d. Ptychography adds the significant benefit of *a posteriori* correction of residual defocus and aberrations. A spherical aberration of the SuperTwin objective lens of $C_S=1.2\,$mm was assumed here. In the reconstructed signal, COF channels are not only visible in the central particle viewed along the COF pores, but also in perpendicular orientation (top left in Fig. 4d). Moreover, the Fourier transform exhibits the present periodicities more clearly than the iDPC$^{(100)}$ signal.

**Figure 5:**
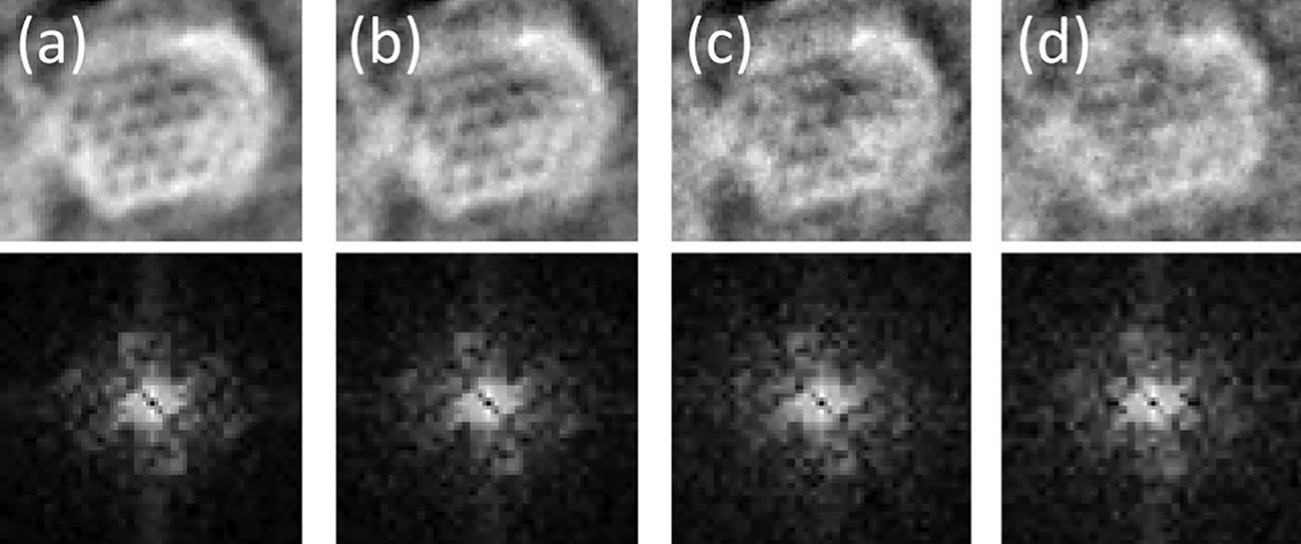
Artificial dose series by data reduction. *Upper row:* iDPC reconstructions employing (**a**) the complete data set of 100 images, and a subset of (**b**) 50 images, (**c**) 25 images or (**d**) 20 images. The dose amounts to 33, 17, 8 and 7 electrons per Å$^2$ at the detector, respectively. It can be seen that some pores are still visible when only a fifth of the experimental data set is used. The lower row shows the corresponding power spectra. Note that this is not strictly equivalent to an experimental dose series as the reciprocal space sampling is reduced simultaneously with the effective dose.

To further demonstrate the low dose capabilities of the method, iDPC reconstructions were performed utilising only a subset of the experimental data set, leading to the images in Fig. 5. The pores of the Kagome structure are still resolved in the iDPC$^{(20)}$ reconstruction using only a fifth of the data set. Here, a dose of seven electrons per Å$^2$ at the detector applies, implying that reliable iDPC results are obtainable for specimen doses between ten and 15 electrons per Å$^2$.

## Discussion

This study demonstrates how momentum-resolved 4D-STEM evaluations can be transferred to conventional, plane wave illumination TEM via the theorem of reciprocity. It was shown that a polar tilt pattern with twelve azimuthal and ten inclination steps, and a precession-based tilt pattern with 100 azimuths and a single inclination are perfectly capable of imaging small built-in electric fields and weakly scattering organic matter.

By using precession for imaging COFs at low-dose and in-focus, the usual measurement time for TEM imaging of one second was retained while increasing image contrast drastically. Importantly, an increase in camera size, i.e., number of pixels and thus imaged area, does not result in longer measurement times. Using advanced cameras from the field of cryo-TEM with $4K\times 4K$ pixels running at 300 Hz frame rate, a 300 segment DPC data set with $4096\times 4096$ image pixels could be recorded in one second. As a comparison a 4D-STEM recording time of 28 min is estimated even with ultrafast diffraction cameras with 10 kHz frame rate.

The sparse data sets require less data to be stored and processed in comparison to 4D-STEM. This represents a major speed advantage and paves the way to enhance live data processing for BF, ABF, DPC, iDPC and direct ptychography straight forwardly at the microscope. Of course, 4D-STEM with pixelated detectors provides comprehensive diffraction information and allows for analysis of dark field and bright field information flexibly by definition of virtual detectors. The full 4D data especially allows for the robust measurement of the angular deflection of the electron beam via COM calculation including both bright and dark field information. The Fourier-based technique is here designed to implement (1) DPC in a parallel illumination setup and therefore concentrates on segmenting the ABF signal. Whereas the COM approach implies that the whole diffraction space should be exploited to obtain an accurate COM signal, the sparsely populated dark field in low-dose studies of weakly scattering objects can lead to less precise COM measurements. In theory, thermal diffuse scattering dominating high angles has the same COM as the elastic scattering^8^, which explains that the *p-n* junction in rather thick GaAs can reasonably be imaged in Fig. 3. However, instead of providing a fully quantitative electric field measurement technique, generating reliable but qualitative contrast at *p–n* junctions was a major motivation here which is expected to have versatile applications in more complex semiconductor nanotechnology devices such as field-effect transistors. Because at low-dose settings, single events at high angles dominate the whole COM calculation due to their weighting with the spatial frequency, determining the COM from diffraction within a cutoff spatial frequency typically improves the signal-to-noise ratio. A 4D-STEM setup also enables advanced processing, e.g., iterative ptychography as with the extended ptychographic iterative engine^11^ (ePIE) or potentially inverse multislice^39,40^. For iterative 4D-STEM ptychography defocusing leads to a larger overlap of probe positions which is beneficial for the deconvolution of specimen and probe. In this study, SSB ptychography has been exploited to correct for aberrations whereas SSB itself yields optimum results at in-focus conditions. To which extent the demonstrated Fourier-based method can be generalised to assure convergence of contemporary iterative, super-resolution phase retrieval schemes needs to be explored in future studies.

The presented method requires no modifications to the microscope. Only two alignment steps are required, centring of the objective aperture and ensuring parallel illumination conditions. In combination with the fact that most contemporary cryo-EMs are rather capable of TEM than STEM, the Fourier-based acquisition schemes can be expected to be beneficial for applications in structural biology where DPC is currently being introduced^14^. In contrast to STEM DPC, the parallel illumination results in a significantly lower required dose since electrons that would transmit undetected through the hole of the DPC detector are excluded already at the illumination step. The local dose rate is an important parameter regarding beam-induced specimen damage, resulting in local dielectric breakdown and possibly bond breaking^41^.

## Conclusion

Acquiring 4D data with combined real and diffraction space information by a tilt series of the incoming beam can significantly enhance the sensitivity for the detection of built-in electric fields and nanostructures made of light atoms. One key advantage of this Fourier TEM approach is that the diffraction space sampling can be tailored continuously, instead of being dictated by detector hardware geometries. The method provides a simple way to introduce differential phase contrast microscopy (DPC) and direct electron ptychography to plane-wave illumination TEM imaging in materials science, soft matter characterisation, and structural biology while rendering more dose-efficient than the STEM based counterpart in many cases.

## Methods

### Acquisition of tilt series in TEM

An FEI Tecnai G2 20 S-TWIN equipped with a TVIPS F216 camera was used for the imaging of the *p–n* junction at 200 keV. The Fourier DPC method was implemented using the TEM Scripting interface supplied by the manufacturer. Images were recorded for each beam tilt setting using a python-based scripting interface supplied by TVIPS with an exposure time of 500 ms. The camera readout, file saving, communication and the TEM scripting incurred an additional overhead of 400 ms per image.

Precession based experiments were performed with an FEI Titan Themis 60–300 equipped with an aberration corrector for the probe, operated at 300 keV. A Quantum Detectors Medipix 3 MerlinEM detector has been employed to record TEM images in continuous mode at 6 bit dynamic range. The microscope was operated in dynamic conical dark field mode, allowing for precession frequencies of up to 10 Hz, whereas 1 Hz was used in the present study. Synchronisation of precession angle (azimuth) and image acquisition was implemented by translating the voltage of the AC beam deflection coils into a suitable trigger for the MerlinEM controller by a voltage threshold based circuitry.

For the reciprocity relation to apply, ensuring parallel illumination as well as objective aperture centring is crucial. Parallel illumination can be realised straight forwardly by adjusting the condenser lens system such that, in diffraction mode, the primary beam and Bragg spots adopt discrete peak shape when observed in the back focal plane of the objective lens. To ensure this, the diffraction focus was tuned such that the rim of an inserted objective aperture appeared sharp in diffraction mode. Due to a possible deviation of the physical objective aperture position from the back focal plane of the objective lens this scheme can lead to slightly converging or diverging illumination, the effect of which is elaborated in simulations in Suppl. Sect. 2. Supplementary Fig. 6 shows that even a large non-isoplanatism of 0.3 mrad yields practically the same iDPC results for reasonable sizes of the illuminated area. Objective apertures of 2.8 mrad in the Tecnai and 6 mrad in the Titan experiments were centred manually around the centre of the tilt pattern. The origin of the coordinate frame for the beam tilt was set to the aperture centre at the optical axis.

Calibration of the aperture sizes and centering was performed using diffraction patterns of GaAs for the Tecnai and Au for the Titan experiments (see Suppl. Sect. 4). The apertures were then used to calibrate the beam tilts. In the Tecnai experiments, a small ellipticity in the beam tilt coordinate system was corrected during analysis. For the precession experiments, the dynamic dark field alignments of the Titan microscope were performed to remove the ellipticity of the beam precession by assuring that the primary beam precesses exactly on the aperture edge in diffraction mode.

### Specimen details

1. *p–n* junction.
  The GaAs crystal was grown using metal-organic vapor-phase epitaxy (MOVPE) on a GaAs substrate. Doping was achieved using carbon in the *p* region and tellurium in the *n* region with the *n* side being doped higher than the *p* side. The *p–n* junction was grown as reported previously^4^, in between two 4 nm thick AlAs marker layers which were spaced far enough apart to not influence the *p–n* junction. The dopant concentration was investigated using secondary ion mass spectrometry (SIMS), Hall and ECV. For the *p* side carrier concentrations of ${8.1}\times 10^{18}\,{{\textrm{cm}}^{-3}}$ (SIMS), ${6.3}\times 10^{18}\,{{\textrm{cm}}^{-3}}$ (Hall) and ${4.8}\times 10^{18}\,{{\textrm{cm}}^{-3}}$ (ECV) were found. On the *n* side the carrier concentrations were determined to be ${2.1}\times 10^{19}\,{{\textrm{cm}}^{-3}}$ (SIMS), ${2.1}\times 10^{19}\,{{\textrm{cm}}^{-3}}$ (Hall) and ${7.8}\times 10^{18}\,{{\textrm{cm}}^{-3}}$ (ECV). As expected SIMS suggests a higher dopant concentration than the ECV measurement as SIMS measures all impurities while Hall and ECV only measure electrically active dopants.
  Specimen preparation was performed using a JEOL JIB 4601 FIB with final polishing at 900 V in a Fischione 1040 NanoMill. TEM imaging was performed with the crystal orientated close to the [110] zone axis.
2. Covalent organic framework (COF).
  The synthesis of the DBC-based COF with imine linkages was performed under argon atmosphere. DBC based node (5.20 mg, 7.5 µmol) and a linear linker (9.04 mg, 15 µmol) were filled into a 6 mL pyrex tube, followed by addition of benzyl alcohol (334 $\upmu$L), mesitylene (166 $\upmu$L), and 6 M acetic acid (50 $\upmu$L). The tube was sealed and heated at 120 $^{\circ }C$ for 3 days. After cooling to room temperature, the precipitate was collected by filtration and Soxhlet extraction with anhydrous tetrahydrofuran was performed for 12 h, yielding a black powder.

### Processing of Fourier DPC data

The calculation of the DPC vector field was performed in full analogy to the approach of STEM DPC imaging. For a single ring of evenly spaced beam tilts the reciprocal space vector is defined as $\vec {k}_\perp = (k_x, k_y)$. In the precession-based approach, this leads to

$$\begin{aligned} I^{\textrm{DPC}}_x (\vec {r})&= \sum _i k_{x,i} I^{\textrm{TEM}}_i (\vec {r})\\ I^{\textrm{DPC}}_y (\vec {r})&= \sum _i k_{y,i} I^{\textrm{TEM}}_i (\vec {r}) \end{aligned}$$

for the *x*- and *y*-components of the DPC vector field, respectively. The sum of the intensities of the TEM images $I^{\textrm{TEM}}_i (\vec {r})$ is normalised to one for each real space pixel. If multiple rings are used, special care is needed for the normalisation and accurate weighting of each micrograph. A derivation of the formula used in the multi-ring case is given in Suppl. Sect. 3.

Integration of the DPC vector field to obtain the iDPC images was performed in Fourier space^25^. The rotation between tilt and real space coordinates was determined by minimising the difference between the original DPC vector field and the field obtained after differentiation of the resulting iDPC, assuming an underlying conservative projected field. A Gaussian high pass filter was added after integration to suppress noise stemming from the low signal-to-noise ratio of low spatial frequencies, a procedure common in iDPC imaging^42^.

The single-sideband (SSB) method^10^ is especially suited for the application to Fourier DPC as it does not require inverse Fourier transforms of diffraction patterns. These are difficult to calculate in non-cartesian coordinate systems. As a consequence, SSB can be applied to any arbitrary sampling of the bright-field disk. The SSB algorithm was implemented based on the formulation of Yang et al.^42^, the phase shift was corrected for the third order spherical aberration $C_{\textrm{S}}$ and defocus in the presented reconstruction.

### Supplementary Information

Supplementary Information.

## Data Availability

The data sets generated during the current study are available from the corresponding author on reasonable request.
