# Supplementary material for: Imaging built-in electric fields and light matter by Fourier-precession TEM
Source: Sci Rep. 2024 Jan 15;14:1320. doi: 10.1038/s41598-024-51423-x (PMC10789819; doi:10.1038/s41598-024-51423-x)
Supplement: Supplementary file 1 — Supplementary Information. [file 41598_2024_51423_MOESM1_ESM.pdf]

Supplementary information to:

Imaging built-in electric fields and light matter by Fourier-precession TEM

Contents

Supplementary figures **1**

1 Dose calibration and thickness dependence **5**

2 Effect of non-isoplanatism and lens aberrations **5**

3 Quantification of momentum transfer and DPC approximation **7**

4 Centering of the objective aperture **7**

Supplementary Figures

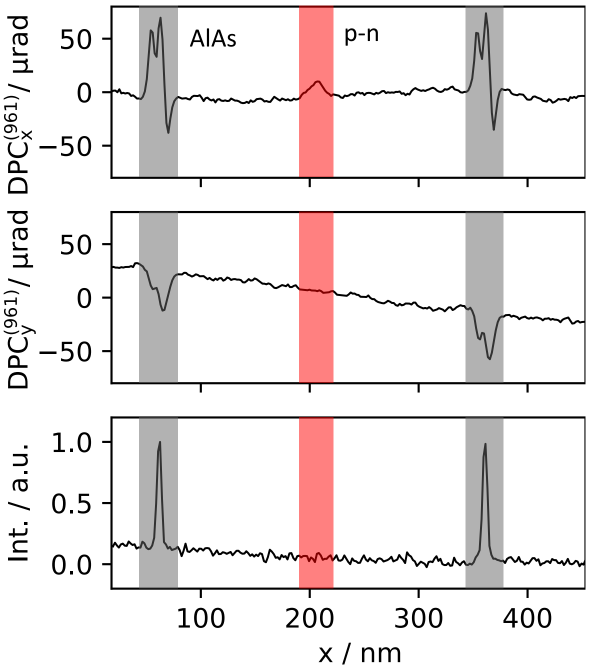

Supplementary Figure 1: **Line profiles across the p-n junction from a cartesian tilt pattern using the Fourier approach.** Averaged COM shifts of the x- and y-components (top, middle) obtained from a 31x31 tilt series arranged in a square pattern as shown in Fig. 3 a. The y-component indicates a thickness- and beam tilt gradient. The TEM intensity (bottom, in arbitrary units). The degradation of the signal due to specimen drift owing to the long acquisition time of over 16 min is especially visible at the interfaces. The area of the p-n junction is highlighted in red, the AlAs layers are marked in grey.

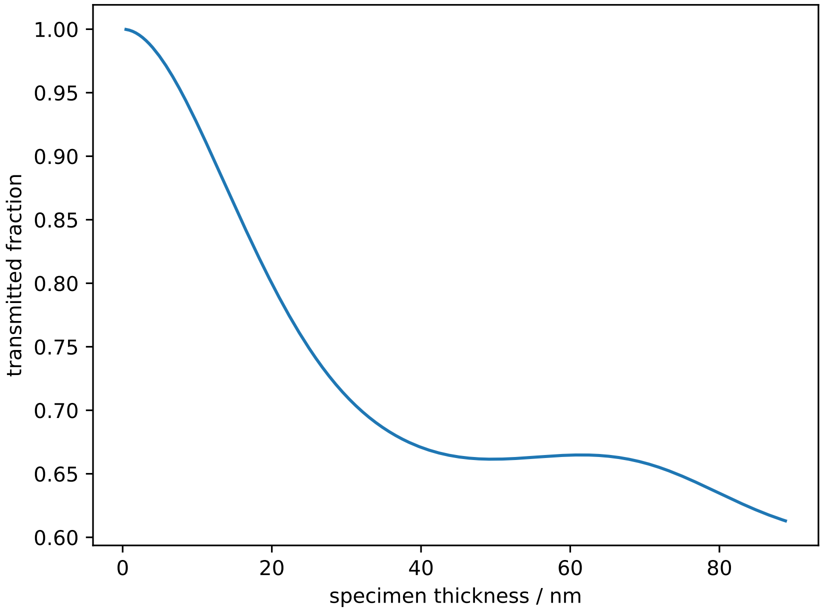

Supplementary Figure 2: Simulation of the transmission through the objective aperture to estimate the dose impinging on the specimen from detector counts. Transmitted fraction of the tilted electron beam calculated for different specimen thicknesses from a multislice simulation. A beam tilt of 5.9 mrad was used in combination with a 6 mrad objective aperture.

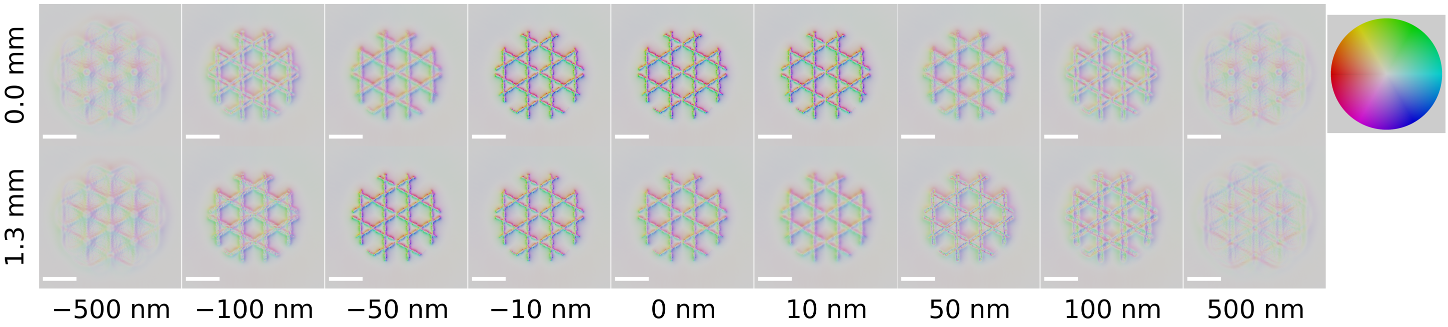

Supplementary Figure 3: Dependence of the DPC signal on focus and spherical aberration (COF simulation). Color-coded vector field calculated for different defoci between −500 and +500 nm (horizontally) without (top) and with spherical aberration $C_S = 1.3$ mm typical for the FEI Super Twin lens (bottom). All plots are presented on the same scale with the used color wheel shown on the top right. The length of the scalebar represents 5 nm.

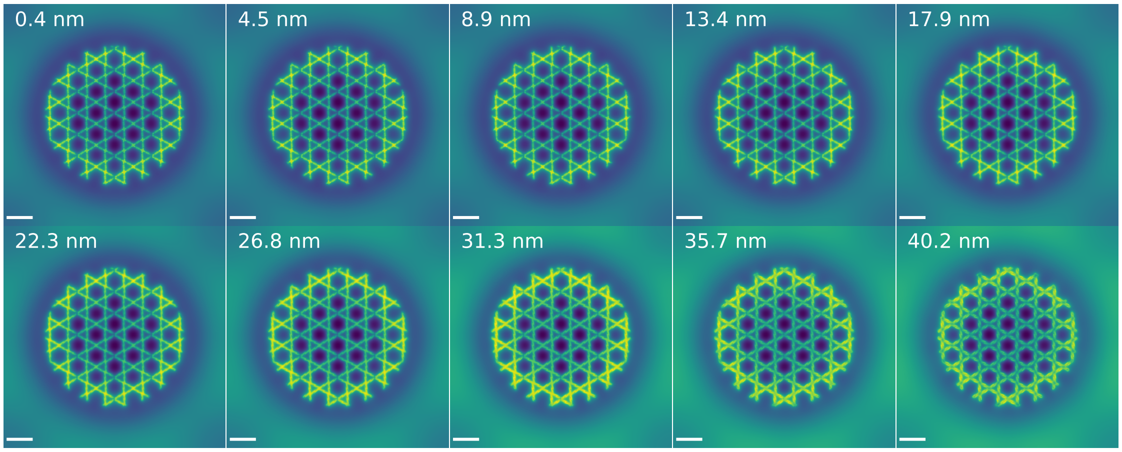

Supplementary Figure 4: **Effect of elevated specimen thickness on iDPC image contrast simulated by multi-slice.** Increasing the specimen thickness leads to a slight blurring of the COF lattice. Note that COF rings tend to stack vertically so as to form elongated tubes. When viewed along the long axis, blurring effects as in the lower row can easily occur due to dynamical scattering and limited depth of field. The thickness is indicated in the top-left corner, the scalebar represents 5 nm.

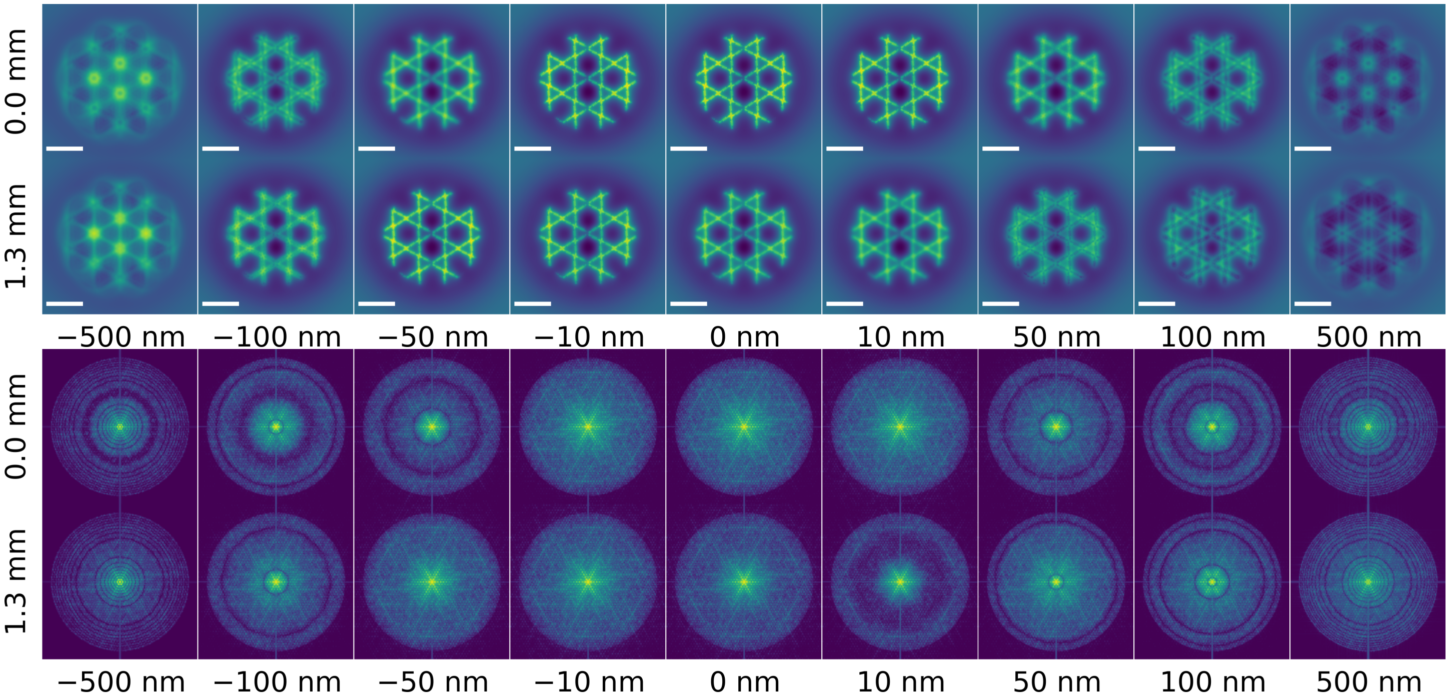

Supplementary Figure 5: **Dependence of the iDPC signal on focus and spherical aberration (COF simulation).** *Top:* The integrated DPC (iDPC) signal was calculated for different defoci between −500 and +500 nm (horizontally) without and with spherical aberration $C_S = 1.3\,\text{mm}$ typical for the FEI Super Twin lens. No high-pass filtering is applied and all images are scaled to the same colorbar. The length of the scalebar represents 5 nm. *Bottom:* Power spectra of iDPC images at the top.

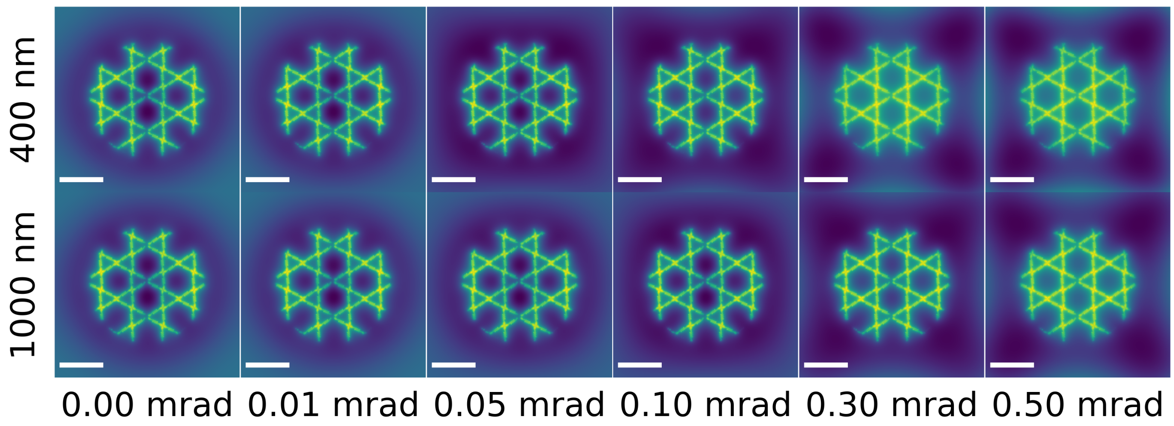

Supplementary Figure 6: **Effect of non-isoplanatism (COF simulation).** Slightly convergent or divergent illumination arises from a deviation of the objective aperture position from the back focal plane of the objective lens, causing a potential misalignment of the condenser. Shown are simulated iDPC images and their power spectra for various non-isoplanatism angles $\theta_{\max} \leq 5\,\mathrm{mrad}$ (horizontal) and illuminated area diameters $r_{\max} = \{1000\,\mathrm{nm},\, 400\,\mathrm{nm}\}$ (vertical). No aberrations were assumed. The length of the scalebar corresponds to $5\,\mathrm{nm}$.

1 Dose calibration and thickness dependence

Dose calibration was performed to relate the number camera counts to the number of detected electrons. The Medipix3 based MerlinEM detector is a hybrid pixel detector where a threshold energy can be selected for each pixel. The datasets in this study were acquired with a threshold of 50 keV. To calibrate the absolute dose in an additional experiment, image series have been recorded under low dose conditions such that the clusters, each of them corresponding to the detection of a single electron, are clearly separated from each other. A clustering procedure was used to determine the number of pixels in each cluster. The mean cluster size then gives the approximate number of counts per electron. For a threshold of 10 keV the average cluster size was determined to 5.8 pixels, whereas the low threshold assured the detection of all electrons as well as possible. Furthermore, Frames with longer acquisition times were recorded for both 10 keV and 50 keV. The average number of counts was 4.254 times higher using the 10 keV setting than the 50 keV setting. From these values it was determined that an electron impinging on the camera produces an average of 1.363 counts under the setting used in the experiment.

The experimentally detected dose at the camera is not equal to the dose the specimen is exposed to because of the objective aperture cutoff in the imaging system. This effect is amplified when beam tilts are introduced and is expected to increase with specimen thickness. To estimate the experimental dose at the specimen multislice calculations were performed for a COF[1] with different thicknesses between 0.4 and 90 nm. The fraction transmitted by through the objective aperture is shown in Supplementary Figure 2. While the portion blocked by the objective aperture increases with specimen thickness, more than 60 % of the intensity passes through the objective aperture even at specimen thicknesses of 80 nm. As a consequence we estimate the complete experimental dose to be at most twice the detected dose.

Using the same multislice simulations the dependence of the iDPC signal on the specimen thickness was investigated for thicknesses between 0.4 and 40.2 nm as shown in Supplementary Figure 4. In the iDPC images elevated specimen thickness results in broadening of the image features. This effect is especially visible when considering the small triangular pores of the Kagome lattice at specimen thicknesses larger than 35.7 nm. Importantly, the specimen features are still recognisable and the simple, though qualitative interpretability of the iDPC images remains valid.

2 Effect of non-isoplanatism and lens aberrations

In TEMs the physical position of the objective aperture can deviate from the back focal plane of the objective lens. This deviation is not only dependent on the exact construction of the lens but also affected by high tension and magnetic hysteresis. Therefore, tuning the diffraction lens such that the objective aperture is in focus and then focusing the primary beam to a spot in the diffraction pattern using the condenser can result in slightly converging or diverging illumination. This effect (non-isoplanatism) will be explored in the following via simulations.

Let us assume that the illuminating wave collapses to a point $\delta(\vec{r})$ on the optical axis at a distance D above or below the specimen, as shown in the schematic below on the left. The wave front $\psi(\vec{r})$ at the specimen plane is then given by a convolution with the Fresnel propagator

$$\psi(\vec{r}) = \iint \delta(\vec{r}')\, \frac{-1}{\mathrm{i}\lambda D} \mathrm{e}^{-\frac{\mathrm{i}\pi}{\lambda D}(\vec{r}-\vec{r}')^2}\, \mathrm{d}^2 r' \tag{1}$$

with the sign in the exponent chosen such that converging illumination is obtained for positive D. Integration immediately yields a parabolic modulation of the illuminating phase

$$\psi(\vec{r}) \propto \mathrm{e}^{-\frac{\mathrm{i}\pi}{\lambda D} r^2} \quad , \tag{2}$$

depending on the squared distance to the optical axis. The effect decreases with increasing distance between specimen and source image with the intended parallel illumination as the limiting case. This results in local beam tilt gradients in the illuminated area which is characterised by the non-isoplanatism angle [2] θ as shown on the right in the schematic below. In the following this effect will be characterised by the maximum non-isoplanatism angle $\theta_{\max}$ at the edge of the illuminated area with a radius $r_{\max}$.

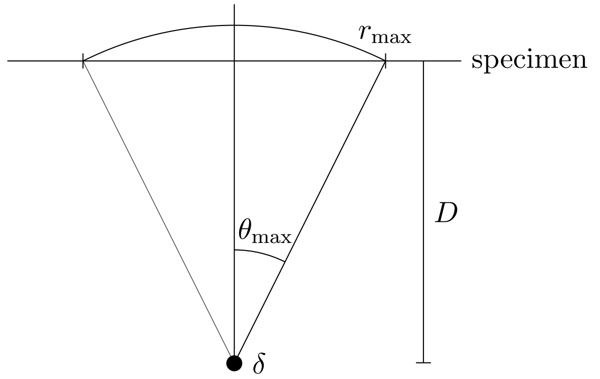

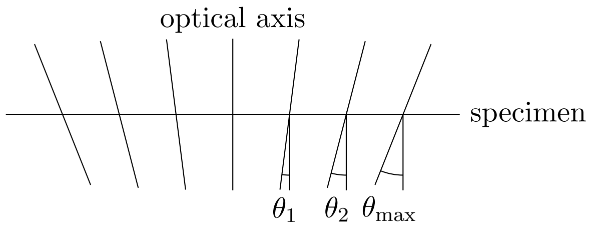

If non-isoplanatism $\theta_{\max}$ and radius of the illuminated area $r_{\max}$ are given, the distance D reads

$$D = \frac{r_{\max}}{\tan \theta_{\max}} \quad . \tag{3}$$

To investigate the effect of this non-isoplanatism on Fourier-DPC experiments, image simulations in the phase object approximation were performed for BDT-ETTA COF crystal with a diameter of 13 nm and a thickness of 5 unit cells. The structure was obtained from literature[1]. In all cases an objective aperture with a radius of 6 mrad was simulated, Fourier-iDPC calculations were performed for 100 beam tilts on a circle with a radius of 5.9 mrad. The simulations were performed for illumination radii of 400 nm and 1000 nm and $\theta_{\max}$ values between 0 and 0.5 mrad.

The resulting iDPC images are shown in Supplementary Figure 6. For both illuminated areas with a radius of 400 and 1000 nm non-isoplanatism below 50 µrad has no visible effect. For larger values of $\theta_{\max}$ low frequency modulations appear which start to become more pronounced at a non-isoplanatism of 0.1 mrad for 400 nm or 0.3 mrad at 1000 nm.

The used FEI Titan Themis microscope is equipped with a three lens condenser system which allows the illuminated area to be changed while retaining parallel illumination. Following manufacturer specifications parallel illumination is retained for illumination radii between 350 nm and 5.5 µm (50 µm C2 aperture in microprobe mode), the non-isoplanatism should be below 0.5 mrad even when illuminating areas with a radius smaller than 5 nm [3]. At the same time the deviation of the objective aperture from the back focal plane should not be more than 0.5 mm which for an illuminated area with a radius of 1 µm limits the maximum non-isoplanatism to about 66 µrad after the collimation procedure outlined above [3]. Comparison of these values with the image simulations above suggests that the deviation of the objective aperture from the back focal plane of the objective lens is negligible for the used TEM as to the methodology presented in the main article.

The same approach was used to investigate the influence of aberrations. Simulations were performed for a number of foci with and without spherical aberration. The color-coded DPC vector field is shown in Supplementary Figure 3, the calculated iDPC and its powerspectrum is shown in Supplementary Figure 5.

Introducing a defocus results in image blurring and a decrease in overall contrast. This is visible in both the DPC and iDPC signal. However, despite this blurring the overall shape of the COF particle and the Kagome lattice are still recognizable at a foci of ±100 nm. In the DPC vector field fringes start to appear at the particle edges for these foci. Only at much larger defoci of ±500 nm the increased effect of aberrations results in contrast

reversal in the iDPC image. As expected, the effect of the spherical aberration can be partly compensated by a small negative defocus.

3 Quantification of momentum transfer and DPC approximation

The calculation of the expectation value of the lateral momentum operator $\langle\vec{p}\rangle$

$$\langle\vec{p}\rangle = \frac{\int_A \mathrm{d}A\ \vec{p}I(\vec{p})}{\int_A \mathrm{d}A\ I(\vec{p})} \tag{4}$$

is given by the first moment of the convergent beam diffraction pattern $I(\vec{p})$. Here, $\vec{p} = h \cdot \vec{k}$ is the lateral momentum, connected to the spatial frequency vector $\vec{k}$ by the proportionality factor h, the Planck constant. In the strict sense, the solid angle A needs to cover the whole diffraction space. However, previous studies have shown that $\langle\vec{p}\rangle$ converges rapidly once the whole bright field region is incorporated [4]. In other words, the centres-of-mass of the bright field and the thermal diffuse scattering dominating the dark field are identical.

For ring-detector geometries as used in conventional STEM DPC and implemented here as Fourier DPC in TEM mode, the situation is different. The central part of the bright field is not recorded at all, and it is by no means given that it obeys the same centre-of-mass as the annular bright field. It is thus clear that the evaluation of segmented detectors covering only the outer portion of the bright field (e.g. as in Fig. 2 a,b,d) necessarily involves further assumptions.

Considering eq. (4), we can split the integrations into the detected part within the annular bright field region A_{out}, and the region A_{in} close to the optical axis, which is not detected,

$$\langle\vec{p}\rangle = \frac{\int_{A_{\mathrm{out}}} \mathrm{d}A\ \vec{p}I(\vec{p}) + \int_{A_{\mathrm{in}}} \mathrm{d}A\ \vec{p}I(\vec{p})}{\int_{A_{\mathrm{out}}} \mathrm{d}A\ I(\vec{p}) + \int_{A_{\mathrm{in}}} \mathrm{d}A\ I(\vec{p})} \approx \frac{\int_{A_{\mathrm{out}}} \mathrm{d}A\ \vec{p}I(\vec{p})}{\int_{A_{\mathrm{out}}} \mathrm{d}A\ I(\vec{p}) + \int_{A_{\mathrm{in}}} \mathrm{d}A\ I(\vec{p})} \quad . \tag{5}$$

In the numerator of eq. (5), the integration over A_{in} yields a contribution to $\langle\vec{p}\rangle$ that is completely lost in segmented ring detector DPC setups. Nevertheless, it is reasonable to assume that the A_{out} integration dominates the numerator due to the much higher values of $\vec{p}$, leading to the approximation in eq. (5) under the condition that a change of $\langle\vec{p}\rangle$ is caused by an intensity redistribution at the bright field edges.

Here, the contribution of the integral over A_{in} in the denominator deserves special attention. The inner area of the bright field disk does contain a large amount of intensity and makes thus a non-negligible contribution to the denominator of eq. (5). It is reasonable to assume that approximately the same average intensity level is present in recorded and non-recorded parts of the bright field disk. One can gauge this average intensity by calculating the signal falling on the detectors recording the bright field part, denoted by $A_{\mathrm{out}}^{\mathrm{BF}}$ and being a subset of the TEM tilt series with tilts smaller than the objective aperture radius. The integral over A_{in} in the denominator of eq. (5) can then be replaced via the mean signal in $A_{\mathrm{out}}^{\mathrm{BF}}$, leading to the Ansatz

$$\int_{A_{\mathrm{in}}} \mathrm{d}A\ I(\vec{p}) \approx \frac{A_{\mathrm{in}}}{A_{\mathrm{out}}^{\mathrm{BF}}} \cdot \int_{A_{\mathrm{out}}^{\mathrm{BF}}} \mathrm{d}A\ I(\vec{p}) \quad , \tag{6}$$

with the measured parts of the bright denoted by $A_{\mathrm{out}}^{\mathrm{BF}}$. Equations (5, 6) formed the basis for the evaluations in this paper, whereas naturally, the discrete integration is replaced by a summation over solid angle patches ΔA in practice. Note that this requires a weighting of each image I_i in the Fourier DPC processing not only with the vector $\vec{p}_i$ but also with ΔA_i.

4 Centering of the objective aperture

In the microscope used, while in conical DF mode, the beam was tilted close to the rim of the objective aperture. By activating the beam precession, the objective aperture is straight forwardly centered by ensuring that the beam

is visible along the full azimuth. In the Tecnai microscope the shadow of the objective aperture was centered around the untilted direct beam.

References

[1] T. Sick, A. G. Hufnagel, J. Kampmann, I. Kondofersky, M. Calik, J. M. Rotter, A. Evans, M. Döblinger, S. Herbert, K. Peters, D. Böhm, P. Knochel, D. D. Medina, D. Fattakhova-Rohlfing, T. Bein, *Journal of the American Chemical Society* **2018**, *140*, 6 2085, pMID: 29249151.

[2] T. Gonen, B. L. Nannenga, Cryoem, **2021**.

[3] FEI, Titan – condenser manual.

[4] K. Müller-Caspary, F. F. Krause, T. Grieb, S. Löffler, M. Schowalter, A. Béché, V. Galioit, D. Marquardt, J. Zweck, P. Schattschneider, J. Verbeeck, A. Rosenauer, *Ultramicroscopy* **2017**, *178* 62.
